# Multiple Mutations—A Genetic Marker for Extracapsular Spread in Human Papillomavirus/p16‐Positive Oropharyngeal Carcinoma

**DOI:** 10.1002/lio2.70094

**Published:** 2025-02-04

**Authors:** Raphaela Graessle, Iris Piwonski, Cora Husemann, Karsten Kleo, Deema Sabtan, Achim Franzen, Heidi Olze, Ulrike Erben, Michael Hummel, Annekatrin Coordes

**Affiliations:** ^1^ Department of Otorhinolaryngology ‐ Head and Neck Surgery University Hospital Ruppin‐Brandenburg, Brandenburg Medical School Theodor Fontane (MHB) Neuruppin Germany; ^2^ Faculty of Health Sciences Brandenburg, Joint Faculty of the University of Potsdam Brandenburg University of Technology Cottbus‐Senftenberg and Brandenburg Medical School Potsdam Germany; ^3^ Department of Otorhinolaryngology ‐ Head and Neck Surgery RWTH Aachen University Aachen Germany; ^4^ Department of Pathology, Charité—Universitätsmedizin Berlin, Freie Universität Berlin Humboldt‐Universität zu Berlin, and Berlin Institute of Health Berlin Germany; ^5^ Department of Otorhinolaryngology, Head and Neck Surgery, Campus Virchow Klinikum and Campus Charité Mitte, Charité—Universitätsmedizin Berlin, Freie Universität Berlin Humboldt‐Universität zu Berlin, and Berlin Institute of Health Berlin Germany

**Keywords:** extracapsular spread, HPV, oropharyngeal carcinoma, PTEN, somatic mutations

## Abstract

**Background:**

In the 8th edition of the TNM classification, extracapsular spread (ECS) became a factor in classifying the UICC stage of oropharyngeal carcinomas (OPSCC). We aimed to find genetic markers for ECS and to identify differences between HPV/p16‐positive and HPV/p16‐negative cases.

**Methods:**

We performed targeted next‐generation sequencing on 99 samples of operable OPSCC and a retrospective analysis of clinical data.

**Results:**

We included 55 HPV/p16‐positive and 44 HPV/p16‐negative patients. We found a significant difference between both groups, particularly in TP53 mutation (*p* < 0.001). Among other things, a small primary tumor (*p* < 0.001), no ECS (*p* = 0.026) were identified as predictors for survival. Multiple mutations were associated with an increased incidence of ECS, especially in HPV+/p16+ cases (*p* = 0.017). A mutation in PIK3CA occurred more frequently in nonsmokers, especially in HPV−/p16− patients (*p* = 0.027). A PTEN mutation—which only occurred in HPV+/p16+ tissues—reduced disease‐free survival (DFS, *p* = 0.026).

**Conclusion:**

The presence of multiple mutations in HPV+/p16+ OPSCC was associated with a higher risk of ECS.

**Level of Evidence:**

3

## Graphical abstract

The presence of multiple mutations in HPV+/p16+ OPSCC was associated with a higher risk of ECS. A PTEN mutation—which only occurred in HPV+/p16+ tissues—reduced disease‐free survival (DFS, *p* = 0.026).

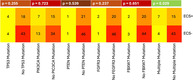

## 1 Introduction

Oropharyngeal and oral cavity carcinomas represent the largest proportion of carcinomas in the head and neck area in Germany [1]. In 2019, oropharyngeal carcinomas (OPSCC) accounted for 37% of carcinomas diagnosed in the head and neck region [2]; 84% of head and neck cancers are squamous cell carcinomas [3]. With the publication of UICC tumor staging (8th edition) in 2017, oropharyngeal squamous cell carcinoma (OPSCC) attracted greater attention. For the first time, cyclin‐dependent kinase inhibitor 2A (commonly abbreviated as p16) status and extracapsular spread (ECS) began to play an important role in the new tumor classification for OPSCC [4, 5]. However, even earlier literature establishes that ECS has a negative impact on overall survival (OS) and disease‐free survival (DFS) [6, 7, 8]. According to the new tumor classification, p16 negative OPSCC with ECS are automatically assigned to the highest lymph node stage (N3b); p16 positive tumors have a better prognosis compared to p16 negative tumors, although they invade the cervical lymph nodes at an earlier stage [9, 10, 11]. Therefore, according to the 8th edition of the UICC tumor classification, it is now possible to assign p16‐positive OPSCC with lymph node infiltration to UICC stage I or II instead of classifying them directly as UICC stage III just because of lymph node infiltration, as was required by the 7th UICC tumor classification. Even though ECS and p16 status are important for tumor prognosis, they currently have limited impact on the treatment of OPSCC patients. A better understanding of the pathomechanisms underlying ECS and p16 positivity could enable more specific and effective treatment. One major problem here is great heterogeneity of OPSCCs, resulting from the variety of risk factors leading to OPSCC such as HPV infection, smoking, alcohol consumption, environmental pollution, and genetic risk factors [12, 13, 14].

Current research is now using next‐generation sequencing (NGS) to identify mutation profiles for various carcinomas. This method has already identified a broad spectrum of genetic changes in HNSCCs that are driver genes for tumor development and influence therapy and prognosis [15, 16, 17]. The use of molecular biomarkers in OPSCC treatment has often been investigated in relatively small studies that included patients with different primary localizations, stages, and treatments [18]. In this study, we attempted to investigate a patient group that was as homogeneous as possible. All included patients suffered from operable OPSCC. Our main objective was to identify genetic markers for ECS in both HPV/p16 positive and negative OPSCC and to determine the influence of mutations on OS and DFS.

## 2 Materials and Methods

### 2.1 Patient Cohort

In this study we included a consecutive cohort of 99 patients with oropharyngeal carcinoma who were initially diagnosed between 2012 and 2020 at the Charité—Universitätsmedizin Berlin and underwent tumor resection. The study was approved by the local ethics committee (EA2/005/18). Each patient underwent the same standard diagnostic procedures. These consisted of a medical history, an examination of the head and neck including an endoscopy, a computer tomography (CT) scan of the neck, thorax and abdomen and a panendoscopy to confirm the histology, rule out a synchronous tumor and diagnose the extent of tumor disease. All patients were subsequently presented to the multidisciplinary tumor conference (consisting of head and neck surgeons, medical and radiation oncologists, pathologists and radiologists).

All clinical data were documented and extracted retrospectively from the electronic patient file. The following clinicopathological variables were recorded: gender (male vs. female), age at initial diagnosis of OPSCC, tobacco exposure (nonsmoker vs. former/current smoker), pack years, alcohol consumption (no ethanol consumption vs. ethanol consumption), recurrence (positive vs. negative), grading (G1 vs. G2 vs. G3), R status (R0 vs. R1), lymphatic invasion (L0 vs. L1), venous invasion (V0 vs. V1), perineural invasion (PNI0 vs. PNI1), T classification (T1‐2 vs. T3‐4), N classification (positive vs. negative), UICC stage (I‐II vs. III‐IV), and ECS (negative vs. positive). The 8th edition of the UICC was applied retrospectively to all prior cases to make them easier to compare. This edition takes into account p16 status and ECS when determining the tumor stage of an OPSCC and has been used regularly at our clinic since 2017. We only included patients whose OPSCC was primarily operable. The goal of each operation was in‐sano resection. All tumor operations were performed transorally where possible. If this was not possible due to the size of the tumors, an external approach was chosen. Each patient underwent ipsilateral or bilateral neck dissection, depending on the tumor stage. If the surgical tumor resection resulted in a pharyngeal defect or functional impairment, a plastic reconstruction was performed using a local or microvascular flap. Adjuvant radiotherapy (RT) was indicated for tumors ≥ T3 and/or histologically proven lymph node involvement. Adjuvant RT was generally performed with 54–66 Gy. In cases with close margins (< 5 mm), ECS or a non‐in‐sano tumor resection (R1), 66 Gy and concomitant chemotherapy were used. Concomitant chemotherapy consisted of cisplatin (5 × 20 mg/m^2^, in the 1st and 5th week of RT, or weekly 30 mg/m^2^) ±5‐fluorouracil (5FU, 5 × 600 mg/m^2^ c.i., the first week of RT).

### 2.2 Immunohistochemistry (IHC) and RNA In Situ Hybridization

The formalin‐fixed and paraffin‐embedded (FFPE) surgical specimens from 99 patients were used to prepare slides for immunohistochemistry.

IHC was performed on tissue microarrays (TMAs) with two cores (1,5 mm diameter) for each case, according to standard procedures. Hematoxylin was used as counterstaining.

For the detection of p16, immunohistochemical staining of the formalin‐fixed and paraffin‐embedded tissue samples was performed using the BenchMark ULTRA Autostainer (Ventana, Tucson, Arizona, USA) and monoclonal rabbit p16 antibodies E6H4 (solution 1:2, Roche/Ventana, Tucson, Arizona, USA) according to the manufacturer's instructions. A positive p16 status was defined by a medium‐to‐strong (2+/3+) intensity of nuclear staining with a distribution of ≥ 75% (of the tumor cells). Cytoplasmatic staining was irrelevant.

To ensure that p16 status matched HPV status, we used an RNA in situ hybridization (RNA‐ISH) test to determine HPV status. High‐risk HPV E6/E7 mRNA was detected, with 18 HPV types recognized: 16, 18, 26, 31, 33, 35, 39, 45, 51, 52, 53, 56, 58, 59, 66, 68, 73, and 82.

We used the RNAscope Probe—HPV‐HR18 assay (Advanced Cell Diagnostics, Newark, CA, USA) according to the instructions. As there were no differences in the results between IHC and RNA‐ISH, we were able to categorize the two groups into HPV+/p16+ and HPV−/p16− subgroups. Conversely, there was no HPV+/p16− group.

### 2.3 DNA Extraction and Next‐Generation Sequencing

gDNA was extracted from unstained tissue sections of each FFPE tissue block using the Maxwell FFPE Plus DNA Kit (Promega, Madison, WI, USA) according to the manufacturer's instructions. For quality assurance purposes, the tumor cell content of each examined tissue sample was counted. The range of tumor cell content was between 5% and 90%. Library preparation was performed using the automated Chef‐ready Cancer Hotspot Panel v.2 according to the manufacturer's instructions (Thermo Fisher Scientific, Waltham, MA, USA) with optimally 50 ng gDNA as input material (lowest 10 ng, see Table S1). For sequencing, 16 libraries were pooled with a final library pool concentration of 35 pM and sequenced on the Ion S5 XL with Ion 530 Chips (Thermo Fisher Scientific, Waltham, MA, USA). BAM files were analyzed with JSI Sequence Pilot, SeqNext V5.4.0 (JSI Medical Systems GmBH, Kippenheim, Germany) and hg19 as reference genome. Publicly available databases (cBioPortal, ClinVar, dbSNP) were used for pathogenicity and knowledge status. Benign and likely benign variants were excluded from downstream analyses [19, 20, 21, 22, 23]. The hotspot regions of the following genes were of particular interest: AKT1, APC, EGFR, ERBB4, FBXW7, FGFR3, HRAS, IDH2, JAK3, KRAS, MET, NRAS, NOTCH1, PIK3CA, PTEN, RB1, RET, SMAD4, TP53, VHL; 83.5% of the mutations identified were pathogenic or likely pathogenic, and 16.5% were categorized as variants of unclear significance. We did not differentiate among those variants.

### 2.4 Statistical Analysis

We analyzed the data set with IBM SPSS Statistics version 26.0.0.0 for macOS (IBM Corp., Armonk, NY, USA) and reported the results considering the sample guidelines [24]. For normally distributed metric variables (age of initial diagnosis), the mean and standard deviation were calculated. For non‐normally distributed metric variables (pack years), the median with range was calculated. We conducted an exploratory data analysis, and all *p* values were reported without adjustment for multiple testing.

For group comparisons with dichotomic variables, the chi‐square test and the Fisher's exact test were used. Metric normally distributed variables were compared using the *T*‐test, metric non‐normally distributed variables using the Mann–Whitney *U*‐test.

To identify clinicopathological variables or different mutations as predictors of OS, the Kaplan–Meier method with survival curves and the log‐rank test were used. For multivariate analyses of OS and DFS, the Cox proportional hazards model was used. The variables considered in this analysis for OS were age at initial diagnosis of OPSCC, T classification, and presence of ECS. In the Cox proportional hazards model for DFS, the variables T classification and HPV/p16 status were considered. OS was defined as the time between the initial staging of OPSCC and the date of death or last follow‐up. DFS refers to the time between the initial diagnosis of the OPSCC and the time of recurrence, death, or last follow‐up. For all tests, *p* values < 0.05 were considered statistically significant.

## 3 Results

### 3.1 Patient Characteristics

This study included 99 patients (mean age 62 years), 55 of whom were HPV+/p16+ (IHC and RNA‐ISH positive for p16/HPV) and 44 HPV−/p16− (IHC and RNA‐ISH negative for p16/HPV). The different clinicopathological factors evaluated are listed in Table 1.

**TABLE 1:** Patient and tumor characteristics of the study population of the OPSCC according to their HPV/p16 status.

Variable	Total	HPV+/p16+	HPV−/p16−	*p*
	*N* = 99	*N* = 55	*N* = 44	
Sex—*n* (%)				**0.006**
Female	27 (27.3)	9 (16.4)	18 (40.9)	
Male	72 (72.7)	46 (83.6)	26 (59.1)	
Age at initial diagnosis of HNSCC, years				0.509
mean (SD)	62 (10)	62 (10)	63 (10)	
Alcohol consumption—*n* (%)^a^				**0.001**
No	40 (72.7)	22 (95.7)	18 (56.3)	
Yes	15 (27.3)	1 (4.3)	14 (43.8)	
Smoking habits—*n* (%)^a^				0.367
Former/current smoker	66 (76.7)	32 (72.7)	34 (81.0)	
Non‐smoker	20 (23.3)	12 (27.3)	8 (19.0)	
Pack years				0.116
Median (range)	30 (100)	30 (100)	34 (70)	
Recurrence—*n* (%)				0.110
Positive	28 (28.3)	12 (21.8)	16 (36.4)	
Negative	71 (71.7)	43 (78.2)	28 (63.6)	
OPSCC characteristics				
Grading—*n* (%)				^b^
G1	1 (1.0)	0 (0)	1 (2.3)	
G2	64 (64.6)	31 (56.4)	33 (75.0)	
G3	34 (34.3)	24 (43.6)	10 (22.7)	
R status—*n* (%)				0.641
0	86 (86.9)	47 (85.5)	39 (88.6)	
1	13 (13.1)	8 (14.5)	5 (11.4)	
L status—*n* (%)				0.951
0	83 (83.8)	46 (83.6)	37 (84.1)	
1	16 (16.2)	9 (16.4)	7 (15.9)	
V status—*n* (%)				0.653^c^
0	94 (94.9)	53 (96.4)	41 (93.2)	
1	5 (5.1)	2 (3.6)	3 (6.8)	
PNI status—*n* (%)^a^				0.241^c^
0	89 (92.7)	50 (96.2)	39 (88.6)	
1	7 (7.3)	2 (3.8)	5 (11.4)	
T classification—*n* (%)				0.576
T1‐2	79 (79.8)	45 (81.8)	34 (77.3)	
T3‐4	20 (20.2)	10 (18.2)	10 (22.7)	
N classification—*n* (%)				**0.023**
*N* > 0	74 (74.7)	46 (83.6)	28 (63.6)	
UICC stage (8th edition)—*n* (%)				**< 0.001**
I‐II	77 (77.8)	52 (94.5)	25 (56.8)	
III‐IV	22 (22.2)	3 (5.5)	19 (43.2)	
ECS—*n* (%)^d^				0.560
Negative	48 (64.9)	31 (67.4)	17 (60.7)	
Positive	26 (35.1)	15 (32.6)	11 (39.3)	

*Note:* Bold indicates statistically significant *p* values < 0.05.

Abbreviations: ECS, extracapsular spread; *N*, number; OPSCC, oropharyngeal squamous cell carcinoma; SD, standard deviation; UICC, Union for International Cancer Control.

^a^ Alcohol consumption: 44 Cases were unknown, 32 of these were HPV+/p16+ and 12 were HPV/p16−; smoking habits: 13 cases were unknown, 11 of these were HPV+/p16+ and two were HPV/p16−; PNI: 3 cases were unknown, all these were HPV+/p16+.

^b^ The requirements to perform a chi‐square test were not fulfilled.

^c^ Fisher's exact test.

^d^ *N* = 74 for all cases with lymph node metastasis.

The majority of patients were male (72.7%, *n* = 72 of 99) and were current or former smokers (76.7%, *n* = 66 of 86). 27.3% of patients regularly consumed alcohol. Most of the OPSCC examined were locally limited tumors (T1‐2, 79.8%, *n* = 79 of 99) with lymph node involvement (N+) in 74.7% of cases. ECS was found in 26 of 74 (35.1%) cases with lymph node metastases. Approximately one fifth of the study population had advanced stage OPSCC (UICC III‐IV, 22.2%, *n* = 22 of 99). Histologically, the included HNSCCs were mostly G2 (64.6%, *n* = 64 of 99), R0 resected (86.9%, *n* = 86 of 99), with no lymphatic invasion (L0, 83.8%, *n* = 83 of 99), no venous invasion (V0, 94.9%, *n* = 94 of 99), and no perineural invasion (PNI0, 92.7%, *n* = 89 of 96).

The HPV/p16 positive group differed significantly from the HPV/p16 negative group. In our study population, HPV/p16 positive patients were more often male (83.6% vs. 59.1%; *p* = 0.006), consumed alcohol less frequently (95.7% vs. 56.3%; *p* = 0.001), and had a less advanced UICC tumor stage (UICC I‐II, 94.5% vs. 56.8%; *p* < 0.001).

### 3.2 Mutations as Predictors of HPV/p16 Positivity, ECS, and Smoking Status

The following mutations were taken into account: TP53, PIK3CA, PTEN, FGFR3, and FBXW7. All other mutations (AKT1, NOTCH1, IDH, VHL, SMAD4, RB1, MET, RET, JAK3, NRAS, KRAS, HRAS, ERBB4, EGFR, APC) occurred less than three times in the entire study population (see Table S2). The median variant allele frequency (VAF) was generally 12.0% (range 1.5%–86.0%). The allele frequencies of the somatic mutation variants analyzed in greater detail in this study are shown in the Table S3. Multiple mutations were also considered, and defined as simultaneously detected mutations. Cases with only one or no identifiable mutation were grouped into the single mutation category.

**FIGURE 1:**
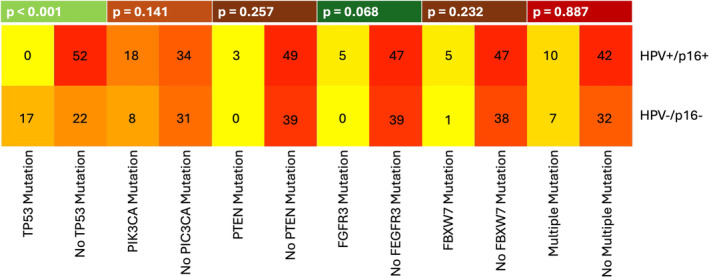
A heatmap showing the most frequent somatic mutations of OPSCC in relation to HPV/p16 status. The *p* value was calculated using the chi‐square test and Fisher's exact test.

**FIGURE 2:**
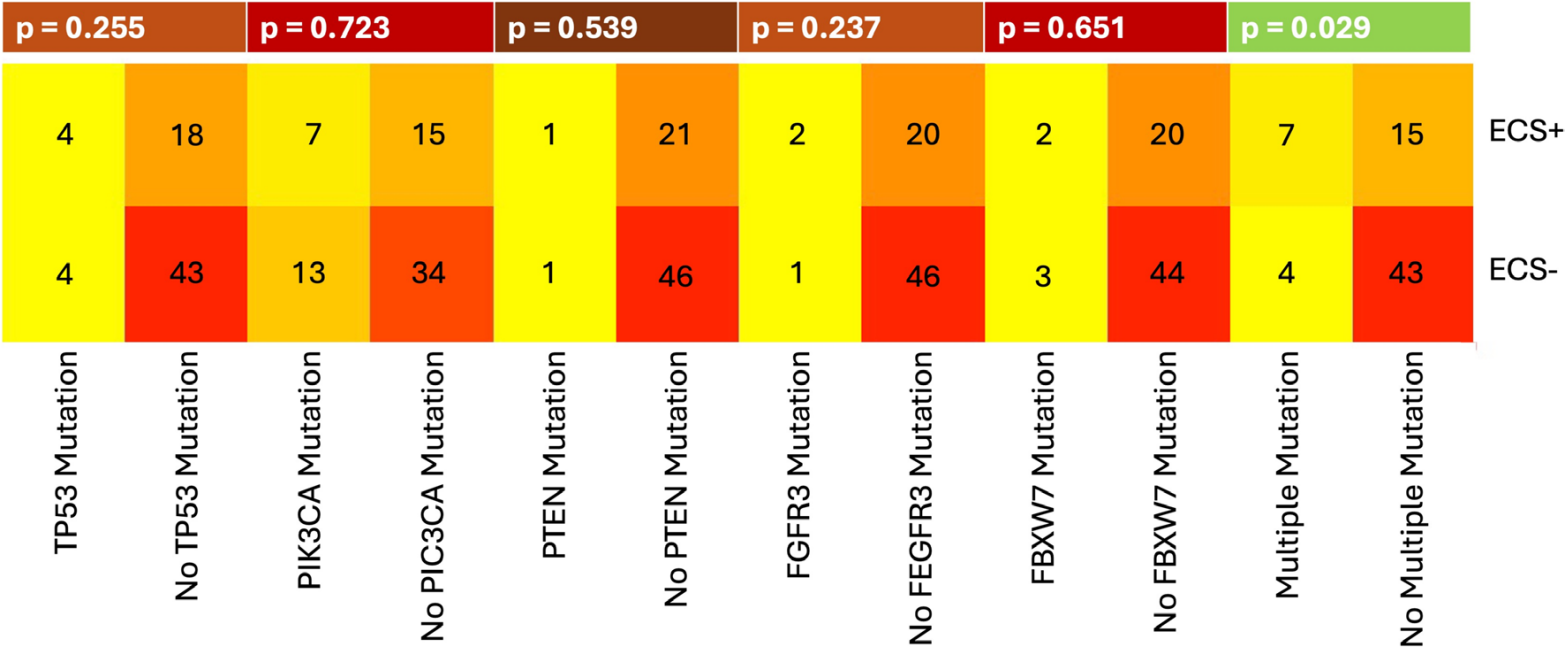
A heatmap showing the most frequent somatic mutations in OPSCC in relation to ECS. The *p* value was calculated using the chi‐square test and Fisher's exact test. ECS, extracapsular spread.

**FIGURE 3:**
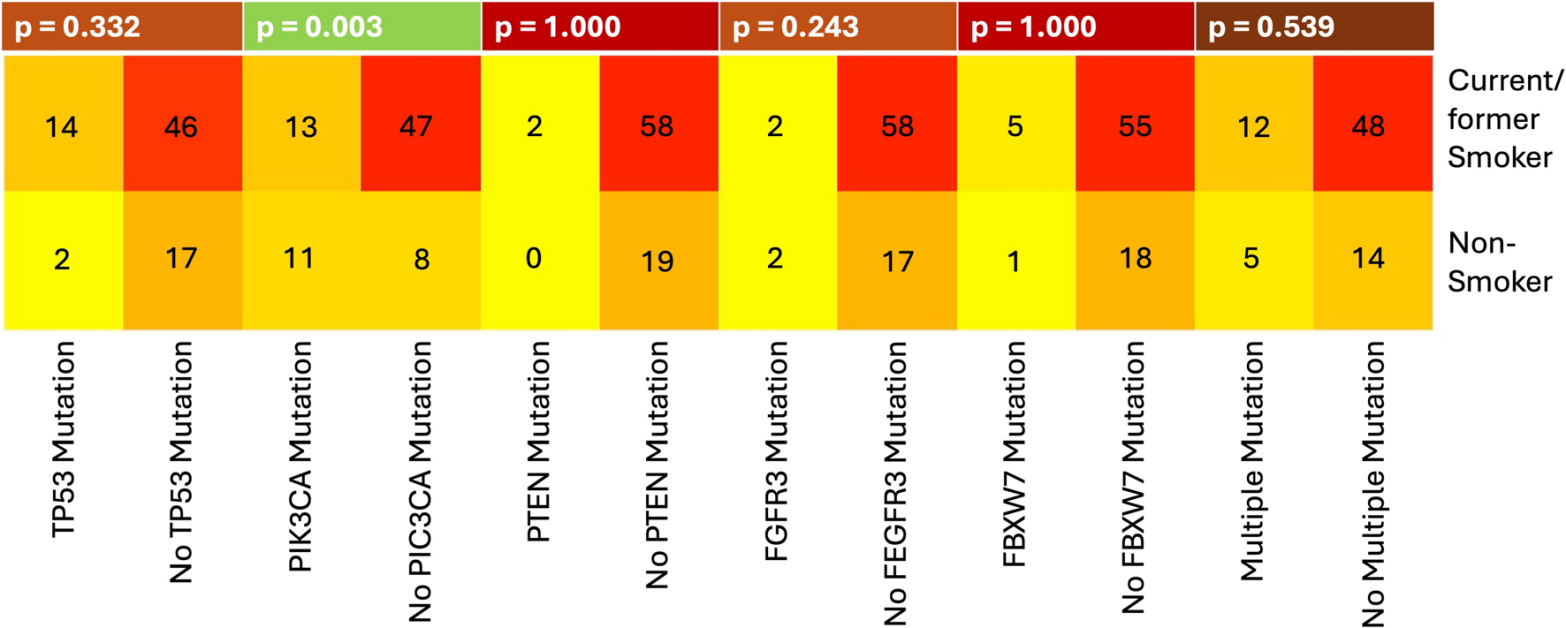
A heatmap showing the most frequent somatic mutations in OPSCC in relation to smoking status. The *p* value was calculated using the chi‐square test and Fisher's exact test.

We correlated patients with somatic mutations of the OPSCC with HPV/p16 status, ECS, and smoking status. Eight samples were excluded due to poor coverage. The results are summarized in Tables S4–S6 and in Figures 1, 2, 3.

We identified 26 patients with PIK3CA mutation in our study population. The second largest mutation group consisted of 17 cases with TP53 mutation, which were all HPV/p16 negative. Accordingly, 17 of the 44 HPV‐negative tumors (38.6%) had a TP53 mutation. This was followed by the group of FBXW7 mutations in 6 cases, the group of FGFR3 mutations in 5 cases and the group of PTEN mutations in 3 cases. The TP53 mutation was a significant predictor of the HPV/p16 status (*p* < 0.001). FGFR3 mutations indicated a correlation with HPV/p16 positivity (*p* = 0.068). FGFR3 mutations only occurred in HPV/p16 positive tumors and the distributions of the PIK3CA, PTEN, FBXW7 or the presence of multiple mutations did not differ between the HPV/p16 negative and HPV/p16 positive carcinomas (*p* = 0. 141; *p* = 0. 257; *p* = 0.232, *p* = 0.877).

In our study population lymph node involvement (N+) with mutations in PIK3CA (*n* = 20), TP53 (*n* = 8), FBW7 (*n* = 5), FGFR3 (*n* = 3) was identified. Patients with multiple mutations suffered from significantly more ECS (*p* = 0.029; see Figure 2 and Table S5). This result can be traced back exclusively to the HPV+/p16+ subgroup (*p* = 0.017, 38.5%, *n* = 5 of 13 multiple mutations in ECS+ tumors versus 6.5%, *n* = 2 of 31 in ECS− tumors). In the HPV−/p16− subgroup, the multiple mutations did not differ as to ECS (*p* = 0.602).

The different mutations analyzed in correlation to smoking status are summarized in Figure 3 (Table S6). The PIK3CA mutation was identified as a predictor for smoking status (*p* = 0.003). PIK3CA mutations were significantly more frequent in nonsmokers (57.9%, *n* = 11 of 19) compared to smokers (21.7%, *n* = 13 of 60). This result can be traced back exclusively to the HPV−/p16− subgroup (*p* = 0.027, *n* = 4 of 7, 57.1% PIK3CA mutations in nonsmokers versus *n* = 4 of 30, 13.3% in smokers). In HPV+/p16+ tumors, PIK3CA mutations and smoking appeared to be unrelated. The TP53, PTEN, FGFR3, FBXW7, or multiple mutations were not associated with smoking status.

### 3.3 Multiple Mutations in Association With the Analyzed Mutations

**FIGURE 4:**
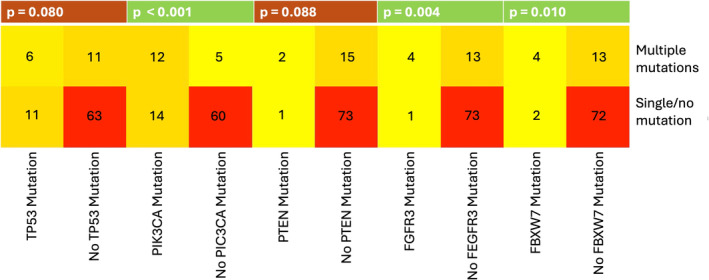
A heatmap showing the most frequent somatic mutations of OPSCC in relation to the presence of multiple mutations. The *p* value was calculated using Fisher's exact test.

We identified 12 PIK3CA, 6 TP53, 4 FGFR3, 4 FBXW7, and 2 PTEN mutations in association with one or more additional mutations detected by the panel. The 17 cases with multiple mutations are listed in Table 2. PIK3CA, FGFR3, and FBXW7 mutations occurred more frequently in OPSCCs with multiple mutations (*p* = < 0.001; *p* = 0.004; *p* = 0.010, see Figure 4, Table S7).

**TABLE 2:** Distribution of multiple mutations.

*N* = 17	HPV/p16	First mutation	Second mutation	Third mutation
1	Negative	TP53	PIK3CA	
2	Negative	TP53	PIK3CA	
3	Negative	TP53	PIK3CA	APC
4	Negative	TP53	JAK3	
5	Negative	TP53	IDH2	
6	Negative	TP53	SMAD4	
7	Negative	PIK3CA	FBXW7	
8	Positive	PIK3CA	FBXW7	
9	Positive	PIK3CA	FGFR3	
10	Positive	PIK3CA	FGFR3	KRAS
11	Positive	PIK3CA	FGFR3	RB1
12	Positive	PIK3CA	NRAS	
13	Positive	PIK3CA	ERBB4	
14	Positive	PIK3CA	MET	
15	Positive	PIK3CA	SMAD4	
16	Positive	PTEN	FBXW7	
17	Positive	PTEN	FBXW7	FGFR3

### 3.4 OS, DFS, and Predictors of OS and DFS

**FIGURE 5:**
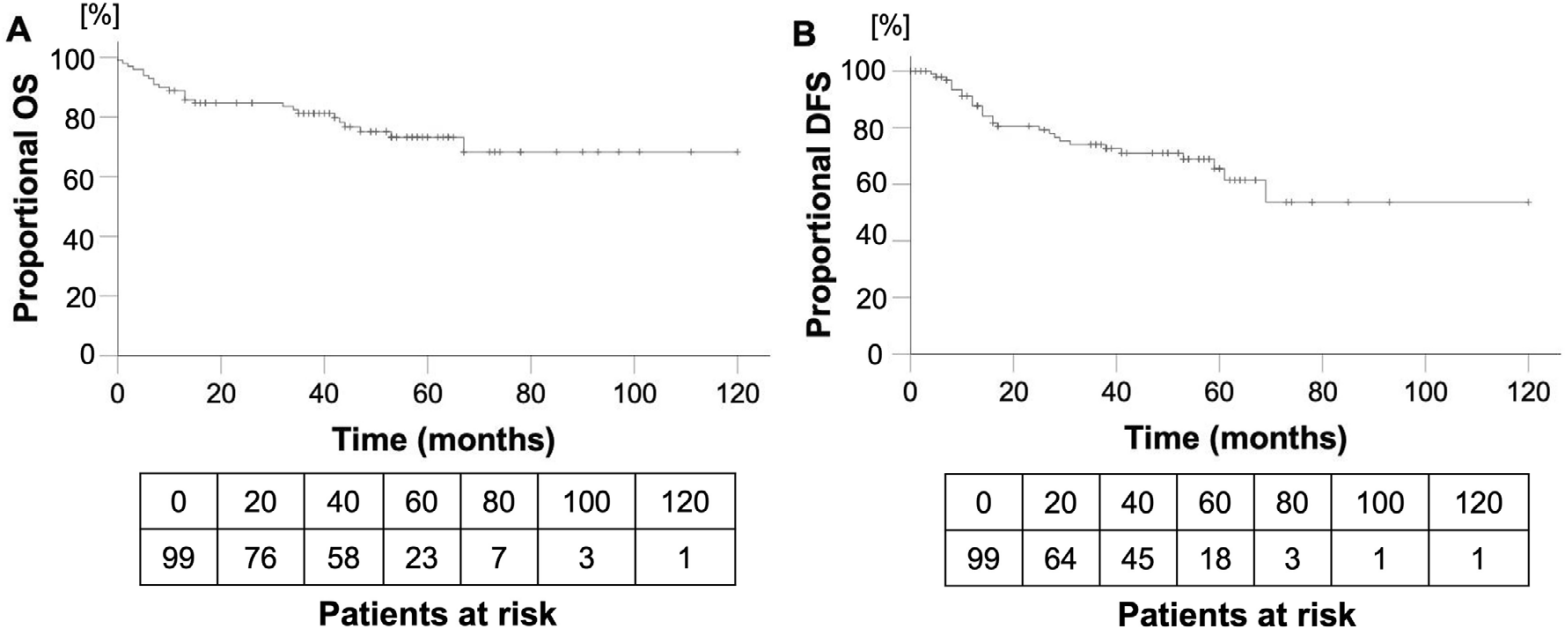
(A) Overall survival (OS) of all 99 patients with oropharyngeal squamous cell carcinoma. (B) Disease‐free survival (DFS) of 99 patients with oropharyngeal squamous cell carcinoma.

In our study population, the mean OS was 91 months (95% CI 81.01–101.48). The 1‐, 3‐ and 5‐year OS were 88.9%, 81.3%, and 73.2%, respectively (Figure 5A). Briefly, 24 (24.2%) patients died during the follow‐up period of up to 120 months (range: 0–120; median 44 months). Four of these deaths were not cancer‐related.

Mean DFS was 81 months (95% CI 67.90–93.24). 1‐, 3‐, and 5‐year DFS rates were 87.7%, 74.0%, and 65.5%, respectively, as shown in Figure 5B; 28 (28.3%) patients suffered a recurrence during the follow‐up period. Among these, 19 (67.9%) patients survived the tumor recurrence and 9 (32.1%) died.

**FIGURE 6:**
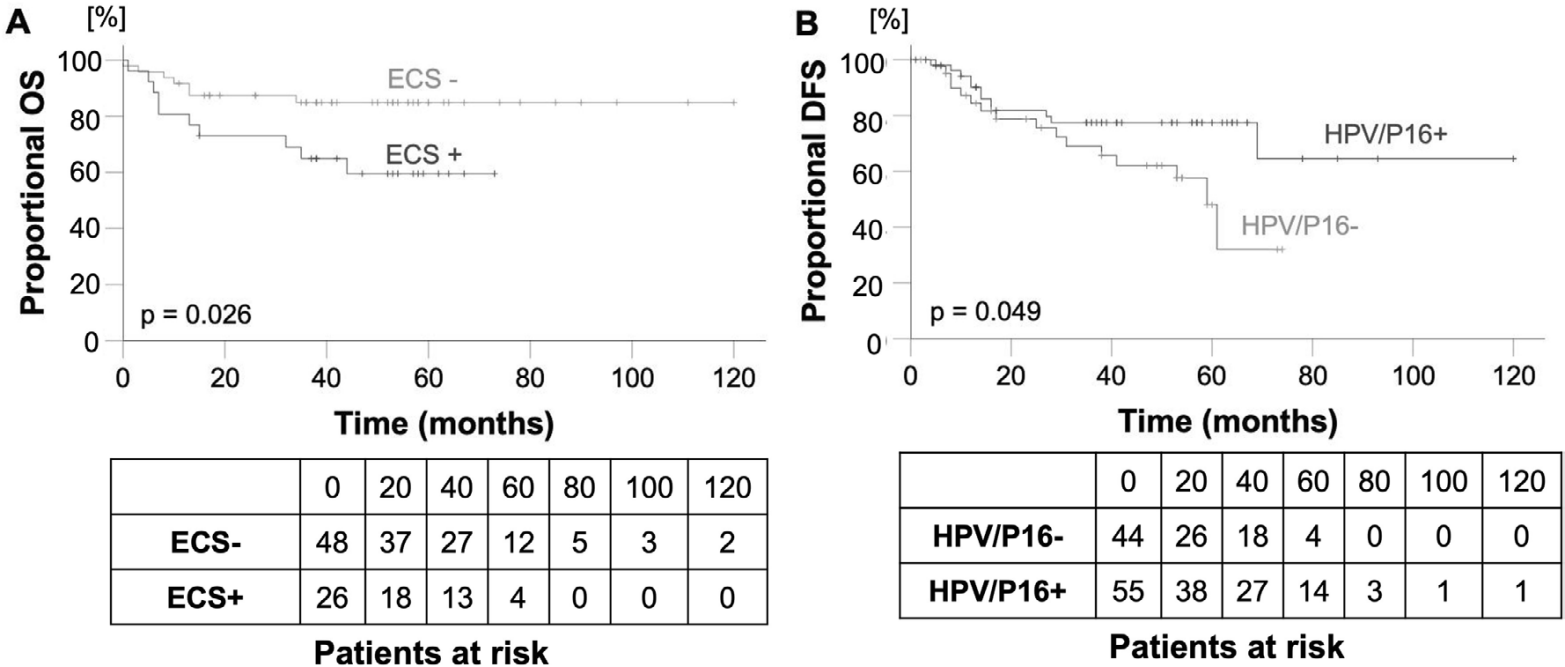
(A) Overall survival (OS) depending on extracapsular spread in oropharyngeal squamous cell carcinoma. ECS+, positive extracapsular spread; ECS−, negative extracapsular spread. (B) Disease‐free survival (DFS) depending on HPV/p16 status in patients with oropharyngeal squamous cell carcinoma. The *p* value was calculated using the log‐rank test.

Among the clinically assessed variables, the univariate analysis showed a significant influence on OS of the following factors: age at initial diagnosis (*p* = 0.029), initial tumor size (T classification; *p* < 0.001), status of venous (*p* = 0.048), and perineural invasion (*p* = 0.008) and ECS (*p* = 0.026, Figure 6A). Being younger at initial diagnosis, a small primary tumor, no venous or perineural invasion and no ECS had a favorable effect on OS (see Table 3). The multivariate analysis also indicated the influence of initial tumor size on OS (*p* = 0.053, hazard ratio (HR) 1.96, confidence interval (CI) 0.993–3.871). The other variables tested in the multivariate analysis showed no significant influence on OS (ECS: *p* = 0.174, HR 2.051, CI 0.727–5.781; age at initial diagnosis: *p* = 0.116, HR 2.191, CI 0.823–5.832).

**TABLE 3:** Univariate analysis of clinicopathologic variables associated with overall survival.

Variable	Total			HPV+/p16+			HPV−/p16−		
	*N* = 99	Mean OS (months/%^a^)	*p*	*N* = 55	Mean OS (months/%^a^)	*p*	*N* = 44	Mean OS (months/%^a^)	*p*
Sex			0.417			0.869			0.145
Female	27	91/76.9		9	89/77.8		18	84/75.6	
Male	72	89/71.4		46	99/78.8		26	58/58.1	
Age at initial diagnosis of HNSCC, years			**0.029**			**0.016**			0.506
≤ 65 years	65	97/79.7		37	108/89.0		28	73/67.3	
> 65 years	34	74/60.0		18	72/55.6		16	53/68.2	
Alcohol consumption^c^			0.753			0.572			0.919
No	40	73/62.7		22	—/64.0		18	72/61.9	
Yes	15	69/70.0		1	—^b^		14	68/68.8	
Smoking habits^c^			0.861			0.599			0.295
Current/former smokers	66	91/69.5		32	94/73.6		34	77/65.5	
Nonsmoker	20	60/73.8		12	68/82.5		8	41/62.5	
Recurrence			0.195			0.242			0.626
Positive	28	76/60.2		12	80/61.1		16	66/57.4	
Negative	71	97/78.8		43	102/83.5		28	58/71.4	
OPSCC characteristics									
Grading			0.275			**0.031**			0.766
G1	1	—^b^		0	—		1	—^b^	
G2	64	—/77.5		31	101/88.6		33	—/67.5	
G3	34	—/64.9		24	85/65.2		10	—/68.6	
R status			0.160			**0.017**			0.738
0	86	93/75.1		47	104/83.5		39	70/65.1	
1	13	73/60.6		8	63/50.0		5	53/80.0	
L status			0.770			0.712			0.971
0	83	92/73.9		46	99/79.3		37	71/66.6	
1	16	52/68.8		9	48/74.1		7	51/64.3	
V status			**0.048**			0.390			**0.045**
0	94	93/75.6		53	99/79.7		41	73/70.4	
1	5	38/30.0		2	46/50.0		3	30/0.0	
PNI status^c^			**0.008**			0.275			**0.020**
0	89	93/74.9		50	98/78.6		39	75/70.5	
1	7	29/38.1		2	35/0.0		5	24/40.0	
T classification			**< 0.001**			**0.008**			0.099
T1‐2	79	100/79.2		45	105/86.5		34	79/69.2	
T3‐4	20	43/47.3		10	40/37.5		10	44/60.0	
N classification			0.775			0.122			0.182
*N* > 0	74	95/75.5		46	—/74.3		28	72/78.4	
*N* = 0	25	75/66.6		9	—^b^		16	58/45.0	
UICC stage (8th edition)			0.860			0.071			0.144
I‐II	77	93/72.2		52	101/81.4		25	66/50.8	
III‐IV	22	69/76.2		3	40/33.3		19	73/84.2	
ECS^d^			**0.026**			**0.009**			0.585
Negative	48	104/84.9		31	106/86.8		17	75/82.4	
Positive	26	51/59.5		15	43/46.9		11	56/72.7	
HPV/p16			0.240			—			—
Negative	55	71/66.6		—	—		—	—	
Positive	44	98/78.5		—	—		—	—	

*Note:* Bold indicates statistically significant *p* values < 0.05.

Abbreviations: ECS, extracapsular spread; OPSCC, oropharyngeal squamous cell carcinoma; OS, overall survival; UICC, Union for International Cancer Control.

^a^ Proportion of patients alive after a follow‐up period of 60 months.

^b^ All cases censored.

^c^ Alcohol consumption: 44 Cases were unknown, 32 of these were HPV+/p16+ and 12 were HPV/p16−; smoking habits: 13 cases were unknown, 11 of these were HPV+/p16+ and two were HPV/p16−; PNI: 3 cases were unknown, all these were HPV+/p16+.

^d^ *N* = 74 for all cases with lymph node metastasis.

Histological grading (*p* = 0.031), R status (*p* = 0.017), and ECS (*p* = 0.009) also significantly influenced OS in the HPV/16 positive group. Both well‐differentiated histology and complete resection of the tumor had a positive effect on OS (see Table 3). No influence on OS was identified for perineural (*p* = 0.275) and venous invasion (*p* = 0.390) in this subgroup. In HPV/p16 negative patients these two variables significantly influenced OS (*p* = 0.020, *p* = 0.045). Additionally, in HPV/p16 negative OPSCC, FBXW7 mutations significantly influenced OS (*p* < 0.001; see Table S8).

DFS was significantly influenced by HPV/p16 status (*p* = 0.049; Figure 6B) and T classification (*p* = 0.026). The multivariate analysis tended to confirm this result (HPV/p16 status: *p* = 0.036, HR 0.443, CI 0.208–0.946, T classification: *p* = 0.056, HR 1.608, CI 0.989–2.615). The remaining clinical variables did not influence DFS in the overall study population or in the HPV/p16 positive subgroups (HPV/p16 +/−). In HPV/p16 negative patients, R0 resection and a small primary tumor were favorable for DFS (see Table S9). PIK3CA and PTEN mutations also indicated a shorter DFS in the total study population (*p* = 0.065; *p* = 0.069). In the HPV/p16 positive subgroup, PTEN mutation had a significant influence on DFS (*p* = 0.026). Not having a PTEN mutation was favorable for DFS (see Table S10).

## 4 Discussion

In this study, we described a selection of somatic mutations in 99 patients (55 HPV+/p16+ and 44 HPV−/p16−) with operable OPSCC and their suitability as predictors for ECS. In accordance with previous studies, ECS was a significant predictor of shorter survival, and our results additionally showed an association between the presence of multiple mutations and ECS [8, 25, 26, 27, 28].

There are many studies that investigate various mutations in head and neck squamous cell carcinomas. The study populations are often small and heterogeneous [15, 18, 29]. In this study, we used a homogenous patient cohort of primary operable OPSCC, which we divided into HPV+/p16+ and HPV−/p16− cases.

The distribution of the recorded clinical characteristics of the patient cohort, including HPV/p16 status, was mostly consistent with literature [30]. However, we found no difference in T classification and smoking habits between HPV+/p16+ and HPV−/p16− patients. The lack of difference in T classification in particular may have been due to our pre‐selection of patients (only operable OPSCC). Our results confirm that a TP53 mutation is one of the most common mutations in HPV−/p16− tumors [15, 31, 32, 33, 34, 35]. Seiwert et al. also found a dominance of FGFR3 mutations in HPV+/p16+ tumors [29]. This study confirmed the results; however, the significance threshold was narrowly missed, probably due to the infrequency of this mutation in our study population.

Regarding the PI3K pathway, the PIK3CA mutation was the most frequent mutation in our study population, followed by the PTEN and AKT1 mutations. These results are in accordance with Cohen et al. [36]. Some other studies have found an association between HPV/p16 positivity and PIK3CA mutations; however, we did not find a significant association in our study population [15, 29, 32].

We have shown that PIK3CA mutations occur significantly more frequently in nonsmoking patients [37, 38]. In contrast, Mirghani et al. did not find an increased incidence of PIK3CA mutations in nonsmokers [39]. We were also unable to detect a higher incidence of PIK3CA in nonsmokers in the HPV+/p16+ OPSCCs subgroup. Although a “nonsmoker” is not clearly defined in the literature, all the studies cited, like ours, considered patients to be nonsmokers if they did not use tobacco regularly in the past or present [37, 38, 39].

In literature, only a small number of publications have investigated ECS and somatic mutations. Sandulache et al. and Gleber‐Netto et al. considered genetic mutations and their association with ECS not in OPSCC, but in oral squamous cell carcinoma [40, 41]. They concluded that a group of TP53 mutations increases the risk of ECS. We were unable to prove a connection between ECS and TP53 with our study population. The reason for this could be that Gleber‐Netto et al. and Sandulache et al. categorized TP53 mutations into high‐risk and low‐risk mutations using the Evolutionary Action scoring system and especially the high‐risk TP53 mutations were associated with ECS [40, 41, 42]. Because of the small number of TP53 mutations in our study, we were unfortunately unable to perform such a grouping and subsequent analysis. In addition, we examined a different tumor location. We investigated OPSCC, whereas a significant association of TP53 and ECS was found in oral squamous cell carcinoma. However, we can confirm that TP53 mutations do not influence clinical outcomes [41]. Park et al. found a disadvantage in survival for the subgroup of patients with TP53 mutated tonsillar carcinoma who underwent adjuvant therapy after tumor surgery [43].

Limitations of this study included the limited number of investigated mutations. We used the Cancer Hotspot Panel V2. The panel is designed to cover 2800 hotspot mutations of 50 oncogenes and tumor suppressor genes. Other studies have already successfully used this panel in connection with head and neck carcinoma [44, 45, 46]. Nevertheless, this panel is not a whole genome sequencing. We attempted to counteract this limitation by combining single mutations and cases in which no mutation could be detected into one group, assuming that at least one mutation, be it identifiable or not, must always be present to allow for carcinogenesis.

Different carcinomas at different sites with mutations in the FBWX gene are associated with a poorer prognosis [47, 48, 49]. We were also able to confirm this in HPV−/p16− OPSCCs with significantly poorer OS.

There are various findings on whether PTEN expression has an effect on the progression of HNSCC [50, 51]. De Kort et al. found that PTEN expression does not affect OS or DFS in HPV−/p16− tumors [50]. Regarding mutations in the PTEN gene, we showed that they do not affect OS. However, PTEN mutations in HPV+/p16+ carcinomas did affect DFS. Whether this is also the case for HPV−/p16− tumors could not be determined in this subgroup due to the lack of detected PTEN mutations. The extent to which the expression is related to the various PTEN mutations could not be clarified in the course of this work. Further research is required to develop a diagnostic tool based on the molecular signatures for ECS. For example, individual mutations associated with ECS can be identified by examining larger study populations. This could lead to a personalized tumor therapy for OPSCC. The use of targeted therapies for adjuvant therapy could then be made dependent on the individual mutation profile.

## 5 Conclusion

In summary, this study showed that multiple mutations in operable OPSCCs are a predictor of ECS in HPV+/p16+ patients. Our study population confirmed earlier published results, indicating that it is representative of the whole population. Future research projects with this population could, for example, focus on the tumor‐specific immune response by investigating cell–cell interactions or transcription factors.

## Ethics Statement

The study was approved by the local ethics committee at Charité—Universitätsmedizin Berlin (EA2/005/18).

## Conflicts of Interest

The authors declare no conflicts of interest.

## Supporting information


**Table S1.** Genes/Amplicons covered by the Cancer Hotspot Panel v2.
**Table S2.** Cross tabulation of the *N* < 3 somatic mutations of OPSCC that have occurred in relation to the HPV/p16 status.
**Table S3.** Variant allele frequency (VAF) of most frequent somatic mutations of OPSCC.
**Table S4.** Cross‐table of the most frequent somatic mutations of OPSCC in relation to the HPV/p16 status.
**Table S5.** Cross‐table of the most frequent somatic mutations in OPSCC in relation to ECS.
**Table S6.** Cross‐table of the most frequent somatic mutations in OPSCC in relation to the smoking status.
**Table S7.** Cross‐table of the most frequent somatic mutations of OPSCC in relation to the presence of multiple mutations.
**Table S8.** Univariate analysis of the most frequent somatic mutations associated with overall survival.
**Table S9.** Univariate analysis of clinicopathologic variables associated with disease free survival.
**Table S10.** Univariate analysis of the most frequent somatic mutations associated with disease free survival.
