# Supplementary material for: Multiple Mutations—A Genetic Marker for Extracapsular Spread in Human Papillomavirus/p16‐Positive Oropharyngeal Carcinoma
Source: Laryngoscope Investig Otolaryngol. 2025 Feb 4;10(1):e70094. doi: 10.1002/lio2.70094 (PMC11791759; doi:10.1002/lio2.70094)
Supplement: Supplementary file 1 — Table S1. Genes/Amplicons covered by the Cancer Hotspot Panel v2. Table S2. Cross tabulation of the N < 3 somatic mutations of OPSCC that have occurred in relation to the HPV/p16 status. Table S3. Variant allele frequency (VAF) of most frequent somatic mutations of OPSCC. Table S4. Cross‐table of the most frequent somatic mutations of OPSCC in relation to the HPV/p16 status. Table S5. Cross‐table of the most frequent somatic mutations in OPSCC in relation to ECS. Table S6. Cross‐table of the most frequent somatic mutations in OPSCC in relation to the smoking status. Table S7. Cross‐table of the most frequent somatic mutations of OPSCC in relation to the presence of multiple mutations. Table S8. Univariate analysis of the most frequent somatic mutations associated with overall survival. Table S9. Univariate analysis of clinicopathologic variables associated with disease free survival. Table S10. Univariate analysis of the most frequent somatic mutations associated with disease free survival. [file LIO2-10-e70094-s001.docx]

**Supp. Table 1:** Genes/Amplicons covered by the Cancer Hotspot Panel v2

| ***ABL1*** |  |
| --- | --- |
| ***AKT1*** |  |
| ***ALK*** |  |
| ***APC*** |  |
| ***ATM*** |  |
| ***BRAF*** |  |
| ***CDKN2A*** |  |
| ***CDH1*** |  |
| ***CSF1R*** |  |
| ***CTNNB1*** |  |
| ***EGFR*** |  |
| ***ERBB2*** |  |
| ***ERBB4*** |  |
| ***EZH2*** |  |
| ***FBXW7*** |  |
| ***FGFR1*** |  |
| ***FGFR2*** |  |
| ***FGFR3*** |  |
| ***FLT3*** |  |
| ***GNA11*** |  |
| ***GNAS*** |  |
| ***GNAQ*** |  |
| ***HNF1A*** |  |
| ***HRAS*** |  |
| ***IDH1*** |  |
| ***IDH2*** |  |
| ***JAK2*** |  |
| ***JAK3*** |  |
| ***KDR*** |  |
| ***KIT*** |  |
| ***KRAS*** |  |
| ***MET*** |  |
| ***MLH1*** |  |
| ***MPL*** |  |
| ***NOTCH1*** |  |
| ***NPM1*** |  |
| ***NRAS*** |  |
| ***PDGFRA*** |  |
| ***PIK3CA*** |  |
| ***PTEN*** |  |
| ***PTPN11*** |  |
| ***RB1*** |  |
| ***RET*** |  |
| ***SMAD4*** |  |
| ***SMARCB1*** |  |
| ***SMO*** |  |
| ***SRC*** |  |
| ***STK11*** |  |
| ***TP53*** |  |
| ***VHL*** |  |
| ***Gene*** | 50 |
| ***Amplicons*** | 207 |

**Supp. Table 2:** Cross tabulation of the N < 3 somatic mutations of OPSCC that have occurred in relation to the HPV/p16 status.

| **Mutation** | | | **Total (%)**  **N = 91** | **HPV+/p16+ (%)**  **N = 52** | **HPV-/p16- (%)**  **N = 39** |
| --- | --- | --- | --- | --- | --- |
| TP53 | | |  |  |  |
|  | TP53 mutation | | 17 (18.7%) | 0 (0%) | 17 (43.6%) |
| PIK3CA | | |  |  |  |
|  | PIK3CA mutation | | 26 (28.6%) | 18 (34.6%) | 8 (20.5%) |
| PTEN | | |  |  |  |
|  | PTEN mutation | | 3 (3.3%) | 3 (5.8%) | 0 (0%) |
| FGFR3 | | |  |  |  |
|  | FGFR3 mutation | | 5 (5.5%) | 5 (9.6%) | 0 (0%) |
| FBXW7 | | |  |  |  |
|  | FBXW7 mutation | | 6 (6.6%) | 5 (9.6%) | 1 (2.6%) |
| AKT1 | | |  |  |  |
|  | AKT1 mutation | | 1 (1.1%) | 1 (2.0%) | 0 (0%) |
| NOTCH1 | | |  |  |  |
|  | NOTCH1 mutation | | 1 (1.1%) | 0 (0%) | 1 (2.6%) |
| IDH2 | | |  |  |  |
|  | IDH2 mutation | | 1 (1.1%) | 0 (0%) | 1 (2.6%) |
| VHL | | |  |  |  |
|  | VHL mutation | | 1 (1.1%) | 1 (2.0%) | 0 (0%) |
| SMAD4 | | |  |  |  |
|  | SMAD4 mutation | | 2 (2.2%) | 1 (2.0%) | 1 (2.6%) |
| RB1 | | |  |  |  |
|  | | RB1 mutation | 2 (2.2%) | 2 (3.8%) | 0 (0%) |
| MET | | |  |  |  |
|  | MET mutation | | 1 (1.1%) | 1 (2.0%) | 0 (0%) |
| RET | | |  |  |  |
|  | RET mutation | | 1 (1.1%) | 1 (2.0%) | 0 (0%) |
| JAK3 | | |  |  |  |
|  | JAK3 mutation | | 2 (2.2%) | 1 (2.0%) | 1 (2.6%) |
| NRAS | | |  |  |  |
|  | NRAS mutation | | 1 (1.1%) | 1 (2.0%) | 0 (0%) |
| KRAS | | |  |  |  |
|  | KRAS mutation | | 1 (1.1%) | 1 (2.0%) | 0 (0%) |
| HRAS | | |  |  |  |
|  | HRAS mutation | | 1 (1.1%) | 1 (2.0%) | 0 (0%) |
| ERBB4 | | |  |  |  |
|  | ERBB4 mutation | | 1 (1.1%) | 1 (2.0%) | 0 (0%) |
| EGFR | | |  |  |  |
|  | EGFR mutation | | 1 (1.1%) | 0 (0%) | 1 (2.6%) |
| APC | | |  |  |  |
|  | APC mutation | | 1 (1.1%) | 0 (0%) | 1 (2.6%) |

**Supp. Table 3:** Variant allele frequency (VAF) of most frequent somatic mutations of OPSCC

| **Mutation** | | ***V*ariant allele frequency [%]**  Median (min.-max., range) |
| --- | --- | --- |
| TP53 mutation | | 23,50 (6.6-86.0, 79.4) |
|  |  |  |
| PIK3CA mutation | | 12,21 (SD 29.50) ^†^ |
|  |  |  |
| PTEN mutation | | 21.35 (7.7-35.0, 27.30) |
|  |  |  |
| FGFR3 mutation | | 18.24 (SD 11.46) ^†^ |
|  |  |  |
| FBXW7 mutation | | 11.00 (9.2-23.0, 13.8) |
|  |  |  |

† Normally distributed, mean (standard deviation); SD, standard deviation.

**Supp. Table 4:** Cross-table of the most frequent somatic mutations of OPSCC in relation to the HPV/p16 status.

| **Mutation** | | | **Total (%)**  **N = 91** | **HPV+/p16+ (%)**  **N = 52** | **HPV-/p16- (%)**  **N = 39** | **p-value** |
| --- | --- | --- | --- | --- | --- | --- |
| **TP53** – n (%) | | |  |  |  | **<0.001** |
|  | No TP53 mutation | | 74 (81.3) | 52 (100) | 22 (56.4) |  |
|  | TP53 mutation | | 17 (18.7) | 0 (0) | 17 (43.6) |  |
| **PIK3CA** – n (%) | | |  |  |  | 0.141 |
|  | No PIK3CA mutation | | 65 (71.4) | 34 (65.4) | 31 (79.5) |  |
|  | PIK3CA mutation | | 26 (28.6) | 18 (34.6) | 8 (20.5) |  |
| **PTEN** – n (%) | | |  |  |  | 0.257^*^ |
|  | No PTEN mutation | | 88 (96.7) | 49 (94.2) | 39 (100) |  |
|  | PTEN mutation | | 3 (3.3) | 3 (5.8) | 0 (0) |  |
| **FGFR3** – n (%) | | |  |  |  | 0.068^*^ |
|  | No FGFR3 mutation | | 86 (94.5) | 47 (90.4) | 39 (100) |  |
|  | FGFR3 mutation | | 5 (5.5) | 5 (9.6) | 0 (0) |  |
| **FBXW7** – n (%) | | |  |  |  | 0.232* |
|  | No FBXW7 mutation | | 85 (93.4) | 47 (90.4) | 38 (97.4) |  |
|  | FBXW7 mutation | | 6 (6.6) | 5 (9.6) | 1 (2.6) |  |
| **Multiple mutations** – n (%) | | |  |  |  | 0.877 |
|  | | No | 74 (81.3) | 42 (80.8) | 32 (82.1) |  |
|  | | Yes | 17 (18.7) | 10 (19.2) | 7 (17.9) |  |

* Fisher's exact test

**Supp. Table 5:** Cross-table of the most frequent somatic mutations in OPSCC in relation to ECS.

| **Mutation** | | | **Total**  **N = 69** | **ECS+**  **N = 22** | **ECS-**  **N = 47** | **p-value** |
| --- | --- | --- | --- | --- | --- | --- |
| **TP53** – n (%) | | |  |  |  | 0.255^*^ |
|  | No TP53 mutation | | 61 (88.4) | 18 (81.8) | 43 (91.5) |  |
|  | TP53 mutation | | 8 (11.6) | 4 (18.2) | 4 (8.5) |  |
| **PIK3CA** – n (%) | | |  |  |  | 0.723 |
|  | No PIK3CA mutation | | 49 (71.0) | 15 (68.2) | 34 (72.3) |  |
|  | PIK3CA mutation | | 20 (29.0) | 7 (31.8) | 13 (27.7) |  |
| **PTEN** – n (%) | | |  |  |  | 0.539^*^ |
|  | No PTEN mutation | | 67 (97.1) | 21 (95.5) | 46 (97.9) |  |
|  | PTEN mutation | | 2 (2.9) | 1 (4.5) | 1 (2.1) |  |
| **FGFR3** – n (%) | | |  |  |  | 0.237^*^ |
|  | No FGFR3 mutation | | 66 (95.7) | 20 (90.9) | 46 (97.9) |  |
|  | FGFR3 mutation | | 3 (4.3) | 2 (9.1) | 1 (2.1) |  |
| **FBXW7** – n (%) | | |  |  |  | 0.651^*^ |
|  | No FBXW7 mutation | | 64 (92.8) | 20 (90.9) | 44 (93.6) |  |
|  | FBXW7 mutation | | 5 (7.2) | 2 (9.1) | 3 (6.4) |  |
| **Multiple mutations** – n (%) | | |  |  |  | **0.029^*^** |
|  | | No | 58 (84.1) | 15 (68.2) | 43 (91.5) |  |
|  | | Yes | 11 (15.9) | 7 (31.8) | 4 (8.5) |  |

* Fisher's exact test

**Supp. Table *6*:** Cross-table of the most frequent somatic mutations in OPSCC in relation to the smoking status.

| **Mutation** | | | **Total (%)**  **N = 79** | **Current/former smoker (%)**  **N = 60** | **Non-smoker (%)**  **N = 19** | **p-value** | **Unknown**  **N = 7** |
| --- | --- | --- | --- | --- | --- | --- | --- |
| **TP53** – n (%) | | |  |  |  | 0.332^*^ |  |
|  | No TP53 mutation | | 63 (79.7) | 46 (76.7) | 17 (89.5) |  | 7 |
|  | TP53 mutation | | 16 (20.3) | 14 (23.3) | 2 (10.5) |  | 0 |
| **PIK3CA** – n (%) | | |  |  |  | **0.003** |  |
|  | No PIK3CA mutation | | 55 (69.6) | 47 (78.3) | 8 (42.1) |  | 6 |
|  | PIK3CA mutation | | 24 (30.4) | 13 (21.7) | 11 (57.9) |  | 1 |
| **PTEN** – n (%) | | |  |  |  | 1.000^*^ |  |
|  | No PTEN mutation | | 77 (97.5) | 58 (96.7) | 19 (100) |  | 6 |
|  | PTEN mutation | | 2 (2.5) | 2 (3.3) | 0 (0) |  | 1 |
| **FGFR3** – n (%) | | |  |  |  | 0.243^*^ |  |
|  | No FGFR3 mutation | | 75 (94.9) | 58 (96.7) | 17 (89.5) |  | 6 |
|  | FGFR3 mutation | | 4 (5.1) | 2 (3.3) | 2 (10.5) |  | 1 |
| **FBXW7** – n (%) | | |  |  |  | 1.000^*^ |  |
|  | No FBXW7 mutation | | 73 (92.4) | 55 (91.7) | 18 (94.7) |  | 7 |
|  | FBXW7 mutation | | 6 (7.6) | 5 (8.3) | 1 (5.3) |  | 0 |
| **Multiple mutations** – n (%) | | |  |  |  | 0.539^*^ |  |
|  | | No | 62 (78.5) | 48 (80.0) | 14 (73.7) |  | 7 |
|  | | Yes | 17 (21.5) | 12 (20.0) | 5 (26.3) |  | 0 |

* Fisher's exact test

**Supp. Table 7:** Cross-table of the most frequent somatic mutations of OPSCC in relation to the presence of multiple mutations

| **Mutation** | | **Total (%)**  **N = 91** | **Multiple mutations (%)**  **N = 17** | **Single/no mutation (%)**  **N = 74** | **p-value** |
| --- | --- | --- | --- | --- | --- |
| **TP53** – n (%) | |  |  |  | 0.080^*^ |
|  | No TP53 mutation | 74 (81.3) | 11 (64.7) | 63 (85.1) |  |
|  | TP53 mutation | 17 (18.7) | 6 (35.3) | 11 (14.9) |  |
| **PIK3CA** – n (%) | |  |  |  | **<0.001^*^** |
|  | No PIK3CA mutation | 65 (71.4) | 5 (29.4) | 60 (81.1) |  |
|  | PIK3CA mutation | 26 (28.6) | 12 (70.6) | 14 (18.9) |  |
| **PTEN** – n (%) | |  |  |  | 0.088^*^ |
|  | No PTEN mutation | 88 (96.7) | 15 (88.2) | 73 (98.6) |  |
|  | PTEN mutation | 3 (3.3) | 2 (11.8) | 1 (1.4) |  |
| **FGFR3** – n (%) | |  |  |  | **0.004^*^** |
|  | No FGFR3 mutation | 86 (94.5) | 13 (76.5) | 73 (98.6) |  |
|  | FGFR3 mutation | 5 (5.5) | 4 (23.5) | 1 (1.4) |  |
| **FBXW7** – n (%) | |  |  |  | **0.010^*^** |
|  | No FBXW7 mutation | 85 (93.4) | 13 (76.5) | 72 (97.3) |  |
|  | FBXW7 mutation | 6 (6.6) | 4 (23.5) | 2 (2.7) |  |

* Fisher's exact test

**Supp. Table 8:** Univariate analysis of the most frequent somatic mutations associated with overall survival.

| **Variable** | | **Total** | | | **HPV/P16+** | | | **HPV/P16-** | | |
| --- | --- | --- | --- | --- | --- | --- | --- | --- | --- | --- |
|  |  | **N = 91** | **Mean OS (months/%**^†^**)** | ***p*-value** | **N = 52** | **Mean OS (months/%**^†^**)** | ***p*-value** | **N = 39** | **Mean OS (months/%**^†^**)** | ***p*-value** |
| **TP53** |  |  |  | 0.088 |  |  |  |  |  | 0.487 |
|  | No TP53 mutation | 74 | 99 (79.7) |  | 52 | 102 (82.3) |  | 22 | 78 (72.7) |  |
|  | TP53 mutation | 17 | 61 (59.4) |  | 0 | - |  | 17 | 61 (59.4) |  |
| **PIK3CA** |  |  |  | 0.126 |  |  | 0.366 |  |  | 0.315 |
|  | No PIK3CA mutation | 65 | 88 (69.7) |  | 34 | 98 (78.7) |  | 31 | 67 (61.0) |  |
|  | PIK3CA mutation | 26 | 70 (88.1) |  | 18 | 72 (88.9) |  | 8 | 64 (87.5) |  |
| **PTEN** |  |  |  | 0.584 |  |  | 0.467 |  |  |  |
|  | No PTEN mutation | 88 | 93 (75.4) |  | 49 | 103 (83.2) |  | 39 | 71 (65.7) |  |
|  | PTEN mutation | 3 | 34 (66.7) |  | 3 | 34 (66.7) |  | 0 |  |  |
| **FBXW7** |  |  |  | 0.920 |  |  | 0.297 |  |  | **<0.001** |
|  | No FBXW7 mutation | 85 | 93 (74.7) |  | 47 | (80.3) |  | 38 | 72 (67.5) |  |
|  | FBXW7 mutation | 6 | 42 (83.3) |  | 5 | - ‡ |  | 1 | 0 (0.0) |  |
| **Multiple Mutations** |  |  |  | 0.820 |  |  | 0.841 |  |  | 0.974 |
|  | No | 74 | 93 (74.9) |  | 42 | 102 (82.9) |  | 32 | 70 (64.5) |  |
|  | Yes | 17 | 62 (76.0) |  | 10 | 66 (80.0) |  | 7 | 53 (71.4) |  |

OS, overall survival.

† Proportion of patients alive after a follow-up period of 60 months.

‡ All cases censored.

**Supp. Table 9:** Univariate analysis of clinicopathologic variables associated with disease free survival.

| **Variable** | | **Total** | | | **HPV/P16+** | | | **HPV/P16-** | | |
| --- | --- | --- | --- | --- | --- | --- | --- | --- | --- | --- |
|  |  | **N = 99** | **Mean DFS (months/%**^†^**)** | ***p*-value** | **N = 55** | **Mean DFS (months/%**^†^**)** | ***p*-value** | **N = 44** | **Mean DFS (months/%**^†^**)** | ***p*-value** |
| Sex |  |  |  | 0.312 |  |  | 0.103 |  |  | 0.566 |
|  | Female | 27 | 52 (65.1) |  | 9 | 53 (66.7) |  | 18 | 48 (64.2) |  |
|  | Male | 72 | 88 (65.8) |  | 46 | 99 (80.1) |  | 26 | 47 (37.2) |  |
| Age at initial diagnosis of HNSCC, years |  |  |  | 0.305 |  |  | 0.167 |  |  | 0.963 |
|  | ≤ 65 years | 65 | 87 (68.9) |  | 37 | 101 (82.1) |  | 28 | 48 (49.7) |  |
|  | > 65 years | 34 | 57 (59.0) |  | 18 | 58 (67.9) |  | 16 | 52 (50.8) |  |
| Alcohol consumption^§^ |  |  |  | 0.858 |  |  | 0.636 |  |  | 0.416 |
|  | No | 40 | 57 (65.3) |  | 22 | (79.4) |  | 18 | 44 (49.2) |  |
|  | Yes | 15 | 57 (67.1) |  | 1 | - ‡ |  | 14 | 56 (64.2) |  |
| Smoking habits^§^ |  |  |  | 0.541 |  |  | 0.279 |  |  | 0.284 |
|  | Current/ former smokers | 66 | 78 (70.0) |  | 12 | 72 (90.9) |  | 8 | 40 (0.0) |  |
|  | non-smoker | 20 | 63 (66.7) |  | 32 | 84 (78.4) |  | 34 | 50 (61.9) |  |
| ***HNSCC characteristics*** |  |  |  |  |  |  |  |  |  |  |
| Grading |  |  |  | 0.644 |  |  | 0.193 |  |  | 0.757 |
|  | G1 | 1 | - ‡ |  | 0 |  |  | 1 | - ‡ |  |
|  | G2 | 64 | (68.6) |  | 31 | 67 (85.8) |  | 33 | (50.2) |  |
|  | G3 | 34 | (56.9) |  | 24 | 84 (65.7) |  | 10 | (32.8) |  |
| R status |  |  |  | 0.116 |  |  | 0.703 |  |  | **0.022** |
|  | 0 | 86 | 86 (67.6) |  | 47 | 95 (76.5) |  | 39 | 52 (54.3) |  |
|  | 1 | 13 | 47 (51.7) |  | 8 | 60 (83.3) |  | 5 | 29 (20.0) |  |
| L status |  |  |  | 0.595 |  |  | 0.595 |  |  | 0.367 |
|  | 0 | 83 | 82 (67.7) |  | 46 | 88 (75.9) |  | 37 | 51 (52.0) |  |
|  | 1 | 16 | 46 (55.7) |  | 9 | 52 (87.5) |  | 7 | 40 (33.3) |  |
| V status |  |  |  | 0.468 |  |  | 0.466 |  |  | 0.101 |
|  | 0 | 94 | 81 (66.6) |  | 53 | (76.5) |  | 41 | 51 (51.4) |  |
|  | 1 | 5 | 43 (50.0) |  | 2 | - ‡ |  | 3 | 28 (0.0) |  |
| PNI status^§^ |  |  |  | 0.056 |  |  | 0.206 |  |  | 0.224 |
|  | 0 | 89 | 81 (65.9) |  | 50 | 90 (77.1) |  | 39 | 51 (50.1) |  |
|  | 1 | 7 | 28 (40.0) |  | 2 | 22 (50.0) |  | 5 | 27 (33.3) |  |
| T classification |  |  |  | **0.026** |  |  | 0.765 |  |  | **0.026** |
|  | T1-2 | 78 | 86 (72.8) |  | 45 | 91 (78.1) |  | 34 | 54 (65.5) |  |
|  | T3-4 | 19 | 41 (31.1) |  | 10 | 48 (75.0) |  | 10 | 34 (16.4) |  |
| N classification |  |  |  | 0.204 |  |  | 0.514 |  |  | 0.522 |
|  | N > 0 | 25 | 58 (51.3) |  | 9 | 65 (62.5) |  | 16 | 44 (30.3) |  |
|  | N = 0 | 74 | 86 (71.4) |  | 46 | 89 (80.4) |  | 28 | 53 (57.2) |  |
| UICC stage (8^th^ edition) |  |  |  | 0.524 |  |  | 0.492 |  |  | 0.367 |
|  | I-II | 77 | 82 (71.7) |  | 52 | 91 (78.0) |  | 25 | 41 (55.0) |  |
|  | III-IV | 22 | 54 (45.1) |  | 3 | 35 (66.7) |  | 19 | 55 (45.6) |  |
| ECS^§^ |  |  |  | 0.461 |  |  | 0.530 |  |  | 0.931 |
|  | Negative | 48 | 88 (75.5) |  | 31 | 91 (82.9) |  | 17 | 53 (58.2) |  |
|  | Positive | 26 | 54 (61.8) |  | 15 | 53 (73.3) |  | 11 | 51 (53.3) |  |
| HPV/p16 |  |  |  | **0.049** |  |  |  |  |  |  |
|  | Negative | 44 | 49 (48.0) |  | - | - |  |  |  |  |
|  | Positive | 55 | 90 (77.5) |  | - | - |  |  |  |  |

OS, overall survival; HNSCC, head and neck squamous cell carcinoma; UICC, Union for International Cancer Control; ECS, extracapsular growth.

† Proportion of patients alive after a follow-up period of 60 months.

‡ All cases censored.

§ The total number does not equal 99 cases.

**Supp. Table 10:** Univariate analysis of the most frequent somatic mutations associated with disease free survival.

| **Variable** | | **Total** | | | **HPV/P16+** | | | **HPV/P16-** | | |
| --- | --- | --- | --- | --- | --- | --- | --- | --- | --- | --- |
|  |  | **N = 91** | **Mean DFS (months/%**^†^**)** | ***p*-value** | **N = 52** | **Mean DFS (months%**^†^**)** | ***p*-value** | **N = 39** | **Mean DFS (months/%**^†^**)** | ***p*-value** |
| **TP53** |  |  |  | 0.318 |  |  |  |  |  | 0.815 |
|  | No TP53 mutation | 74 | 85 (72.7) |  | 52 | 91 (79.1) |  | 22 | 48 (54.9) |  |
|  | TP53 mutation | 17 | 50 (41.3) |  | 0 |  |  | 17 | 50 (41.3) |  |
| **PIK3CA** |  |  |  | 0.065 |  |  | 0.182 |  |  | 0.412 |
|  | No PIK3CA mutation | 65 | 73 (61.0) |  | 34 | 83 (74.0) |  | 31 | 46 (43.4) |  |
|  | PIK3CA mutation | 26 | 68 (79.5) |  | 18 | 71 (88.2) |  | 8 | 58 (55.6) |  |
| **PTEN** |  |  |  | 0.069 |  |  | **0.026** |  |  |  |
|  | No PTEN mutation | 88 | 84 (68.7) |  | 49 | 94 (82.1) |  | 39 | 49 (48.7) |  |
|  | PTEN mutation | 3 | 21 (33.3) |  | 3 | 21 (33.3) |  |  |  |  |
| **FBXW7** |  |  |  | 0.726 |  |  | 0.937 |  |  |  |
|  | No FBXW7 mutation | 85 | 82 (66.8) |  | 47 | 91 (79.0) |  | 38 | (48.7) |  |
|  | FBXW7 mutation | 6 | 43 (80.0) |  | 5 | 43 (80.0) |  | 1 | - ‡ |  |
| **Multiple Mutations** |  |  |  | 0.137 |  |  | 0.367 |  |  | 0.276 |
|  | No | 74 | 77 (63.1) |  | 42 | 88 (76.8) |  | 32 | 47 (41.6) |  |
|  | Yes | 17 | 68 (84.6) |  | 10 | 71 (88.9) |  | 7 | 59 (75.0) |  |

OS, overall survival.

† Proportion of patients alive after a follow-up period of 60 months.

‡ All cases censored.
